# Experimental Gas Gangrene

**Published:** 1919

**Authors:** 


***Experimental Gas Gangrene. The Protection Afforded by Various Anti-sera and Anti-sera Mixtures.*** By MARY NEVIN, A. R. C. Research Laboratory, No 1.

It is generally known that gas gangrene is almost never due to pure B. Welchii infection, but to a combined action of a variety of anerobic organisms. It is further held that B. Welchii is not the most important factor in the etiology of the disease, and that the presence of 2 other anerobic bacilli, B. œdematiens and Vibrion septique, constitutes a much more serious bacteriological finding. A series of experiments was made with a view to supplying the following data :

1) The relative value of Bacillus Welchii antitoxin and perfringens anti-microbial serum.

2) The value of various anti-sera both singly and combined against oedematous exudate produced by the inoculation of mixed anerobic organism.

3) The protective value of various anti-sera both singly and combined against mixed anerobic infections.

Of the animals receiving cultures of anti-perfringens serum, five died in less than 24 hours and one in less than 48. Of the 6 that lived, only one gave any reaction to the culture. This animal developed an extensive necrosis of the abdominal surface.

Of the animals receiving cultures of B. Welchii antitoxin, 3 died within 24 hours, 2 within 48 hours and one within 5 days. Of the remaining 6 animals, 3 developed within 48 hours and one within 6 days a liquefactive necrosis over the abdominal surface, with marked swelling of the inoculated leg. Only 2 of the animals failed to develop any evidence of the inoculation.

From these experiments it would seem that some protection is afforded by both the B. Welchii antitoxin and the anti-perfringens-microbial serum against inoculation of pure cultures of B. Welchii.

The protection established by the anti-perfringens-microbial serum seems to be more clean cut in its results than the protection by the B. Welchii antitoxin.

The antibodies contained in the anti-microbial serum are more effective than the anti-toxic principle contained in the antitoxin in preventing circumscribed proliferation of the organisms, thus eliminating even local necrotic areas.

The production of oedema in the development of gas gangrene is a very frequent occurrence.

A series of experiments was carried out with oedematous fluid collected from animals inoculated with different combinations of anerobes in the following way :

Virulent 18-hour meat cultures of B. Welchii, Vibrion septique and B. sporogenes were mixed in equal quantities and guinea pigs inoculated intra-muscularly with 5 cc. of the mixture. Upon the death of the animals, the fluid was collected and the M. L. D. established.

Guinea pigs weighing from 300 to 400 grams were inoculated as follows : 0.5 cc. per 100 gr. weight, and 4 days later all were inoculated with M. L. D.'s of the oedema.

3 with B. Welchii antitoxin (death within 24 to 48 hours).

3 with anti-perfringens serum (death within 24 to 48 hours).

3 with anti-Vibrion septique serum (1 animal died in 3 days).

3 with aa B. Welchii antitoxin and anti-Vibrion septique serum (1 animal died in 24 hours).

3 with aa anti-perfringens serum and anti-Vibrion septique serum (1 animal died in 55 hours).

3 with normal horse serum (death within 24 hours).

It seems apparent that neither anti-perfringens serum nor Bull's antitoxin afford any protection when other anerobic pathogenic organisms are present together with B. Welchii.

These 2 sera cannot therefore be considered to have any practical value in the treatment of wounds, since B. Welchii, in almost every instance, occurs in association with other anerobes. On the other hand Vibrion septique serum shows evidence that it is applicable in a practical way to war wounds, because it is able to combat alone a mixed infection.

24 hour-cultures of the following organisms were mixed in equal amounts and the 48-hour M. L. D. of the mixtures established.

| Culture I. | Culture II | Culture III |
| --- | --- | --- |
| B. Welchii. | B. Welchii. | B. Welchii. |
| Vibrion septique. | B. oedematiens. | B. bellonensis. |
| B. sporogenes. | B. sporogenes. | B. sporogenes. |

9 guinea pigs were subcutaneously inoculated with the anti-sera given below, 0.5 cc. per 100 gr. body weight.

Those receiving culture I were inoculated as follows :

3 with B. Welchii antitoxin.

3 with anti-perfringens serum.

3 with anti-Vibrion septique serum.

3 with B. Welchii antitoxin and anti-Vibrion septique serum aa.

3 with anti-perfringens serum and anti-Vibrion septique serum aa.

Those receiving culture II were inoculated as follows :

3 with B. Welchii antitoxin.

3 with anti-perfringens serum.

3 with anti-oedematiens serum.

3 with B. Welchii antitoxin and anti-oedematiens serum aa.

3 with anti-perfringens serum and anti-oedematiens serum aa.

Those receiving culture III were inoculated as follows :

3 with B. Welchii antitoxin.

3 with anti-perfringens serum.
3 with anti-bellonensis serum.
3 with B. Welchii antitoxin and anti-bellonensis serum aa.
3 with anti-perfringens serum and anti-bellonensis serum aa.

4 days later the animals were injected with M. L. D.'s of the various culture mixtures.

In no instance was any protection established by either the B. Welchii anti-toxin or the anti-perfringens serum.

In all instances when anti-Vibrion septique, anti-oedematiens or anti-bellonensis serum was used, 100 o/o protection was afforded.

The effectiveness is again demonstrated of anti-Vibrion septique serum alone to combat a mixed infection.

*Conclusions.* 1) In the treatment of pure B. Welchii infection, B. Welchii antitoxin is of less prophylactic value than B. Welchii anti-microbial serum.

2) In Vibrion septique, B. oedematiens or B. bellonensis mixed infection, the use of a specific serum, even when diluted by the addition of other sera, is effective.

3) Neither the B. Welchii anti-microbial serum nor the B. Welchii antitoxin is of any value in the prophylaxis of gas gangrene caused by mixed anerobic infection, such as is commonly found in war wounds to-day.

